# Jia-Wei-Kai-Xin-San Treatment Alleviated Mild Cognitive Impairment through Anti-Inflammatory and Antiapoptotic Mechanisms in SAMP8 Mice

**DOI:** 10.1155/2023/7807302

**Published:** 2023-11-02

**Authors:** Xiaolu Zhang, Yingxin Sun, Qun Yu, Wenyun Zeng, Yue Zhang, Miao Zeng, Kexin Pang, Yifei Yu, Jiali Gan, Huhu Li, Lin Yang, Xijuan Jiang

**Affiliations:** ^1^Tianjin University of Traditional Chinese Medicine, Tianjin 301617, China; ^2^Tianjin University of Sport, Tianjin 301617, China; ^3^Ganzhou People's Hospital, Ganzhou 341000, China; ^4^The University of Warwick, Coventry, West Midlands, UK; ^5^Mcmaster University, Hamilton, ON, Canada

## Abstract

**Background:**

Alleviating mild cognitive impairment (MCI) is crucial to delay the progression of Alzheimer's disease (AD). Jia-Wei-Kai-Xin-San (JWKXS) is applied for treating AD with MCI. However, the mechanism of JWKXS in the treatment of MCI is unclear. Thus, this study aimed to investigate the effect and mechanism of JWKXS in SAMP8 mice models of MCI.

**Methods:**

MCI models were established to examine learning and memory ability and explore the pathomechanisms in brain of SAMP8 mice at 4, 6, and 8 months. The mice were treated for 8 weeks and the effects of JWKXS on MCI were characterized through Morris water maze and HE/Nissl's/immunohistochemical staining. Its mechanism was predicted by the combination of UPLC-Q-TOF/MS and system pharmacology analysis, further verified with SAMP8 mice, BV2 microglial cells, and PC12 cells.

**Results:**

It was found that 4-month-old SAMP8 mice exhibited MCI. Two months of JWKXS treatment improved the learning and memory ability, alleviated the hippocampal tissue and neuron damage. Through network pharmacology, four key signaling pathways were found to be involved in treatment of MCI by JWKXS, including TLR4/NF-*κ*B pathway, NLRP3 inflammasome activation, and intrinsic and extrinsic apoptosis. *In vitro* and *in vivo* experiments demonstrated that JWKXS attenuated neuroinflammation by inhibiting microglia activation, suppressing TLR4/NF-*κ*B and NLRP3 inflammasome pathways, and blocking the extrinsic and intrinsic apoptotic pathways leading to neuronal apoptosis suppression in the hippocampus.

**Conclusion:**

JWKXS treatment improved the learning and memory ability and conferred neuroprotective effects against MCI by inducing anti-inflammation and antiapoptosis. *Limitations*. The small sample size and short duration of the intervention limit in-depth investigation of the mechanisms. *Future Prospects*. This provides a direction for further clarification of the anti-AD mechanism, and provides certain data support for the formulation to move toward clinical practice.

## 1. Introduction

Alzheimer's disease (AD) is the most common type of dementia among the elderly [1]. There is currently no effective treatment for this disease due to its complexity and unclear mechanisms. In AD, a long predementia phase is known as mild cognitive impairment (MCI) [2]. Recent AD guidelines recommend that MCI be viewed as a disorder with cognitive abnormalities that exists between normal aging and AD [3]. MCI incidence among the elderly is increasing continuously in China due to economic advancements and population aging, with an incidence rate of approximately 3%–42% [4]. If untreated, approximately half of MCI patient's progress to dementia does so within 3 years [5]. Assessing and controlling risk factors for conversion from MCI to AD can delay or prevent the onset of disease [6]. Thus, the need for an effective treatment for MCI is urgent.

SAMP8 is a spontaneous animal model characterized by age-associated pathologies in the hippocampus and other brain regions, including A*β* deposits, tau hyperphosphorylation, inflammation, endoplasmic reticulum stress, and neuronal injury [7]. Since all these changes resemble AD pathology, SAMP8 mice can be used as models for studying cognitive decline with aging. The aging-related pathologies could be used to investigate potential anti-AD drugs along the aging process. Due to the different experimental purposes, some studies target the early onset of AD (6–8 weeks of age) and late stages of AD (10–12 months), while most studies target the adult stage (4–6 months of age). Therefore, the primary objective of this study was to use SAMP8 mice with MCI for a pharmacological intervention.

Kai-Xin-San (KXS) is a widely used TCM formula for treating dementia that has been used since the Tang Dynasty. It contains *Ginseng quinquefolium* (L.) Alph.Wood (Renshen), *Polygala tenuifolia* Willd (Yuanzhi), *Acorus tatarinowii* Schott (Shichangpu), and *Poria cocos* (Schw.) Wolf (fuling) in a ratio of 3 : 2 : 2 : 3 [8]. Modern research suggests that KXS reduces oxidative stress and apoptosis, protecting neurons and improving cognitive function [9]. Besides apoptosis and oxidative stress, Guo et al. [10] indicated that KXS treatment significantly improved cognition impairment by decreasing neurotransmitter loss and inhibiting the hyperphosphorylation of Tau and neuroinflammation. Similar findings showed that KXS ameliorated cognitive dysfunction in mice induced with SCOP by inhibiting microglia inflammation [11]. Moreover, *Astragalus membranaceus* (Fisch.) Bunge (Huangqi), *Ligusticum wallichii* Franch (Chuanxiong), and *Cistanche deserticola* Y.C.Ma (Roucongrong) are also commonly used for treating amnesia and dementia [12–14]. Studies showed that Astragalus polysaccharide alleviated cognitive impairment and *β*-amyloid accumulation in APP/PS1 mice [15] and Astragaloside IV ameliorated cognitive impairment and neuroinflammation in an oligomeric A*β* induced AD mice model [16]. Guo et al. [17] found that *Cistanche deserticola* Y.C.Ma had a potential to be a possible treatment option for mild to moderate AD. Additionally, *Ligusticum wallichii* Franch has anti-inflammation and antiapoptosis properties [18], in which ligustrazine markedly inhibited A*β*25–35-induced microglia-mediated inflammatory response [19] and treatment with ligustilide significantly ameliorated memory impairment and A*β* levels [20]. Therefore, according to the compatibility of traditional Chinese medicine and the basis of previous research, we used “KXS” as the basic formula and added “*Cistanche deserticola* Y.C.Ma, *Astragalus membranaceus* (Fisch.) Bunge and *Ligusticum wallichii* Franch” to form “Jia-Wei-Kai-Xin-San (JWKXS)” to enhance its efficacy in this study. Similar to this study, there was a study demonstrated that JWKXS improved cognitive deficit in mice with A1–42-induced cognitive deficit by lowering A*β* levels, defending neurons from oxidative damage, and nourishing neurons [21]. Thus, JWKXS may be considered a valuable candidate for MCI therapy.

Due to the multiple ingredients, targets, and pathways of TCM, it can be challenging to use experimental methods to explain its potential mechanisms. As a new methodological system, network pharmacology offers the potential for exploring the efficacy of TCM based on pharmacology and pharmacodynamics [22]. This study evaluated the efficacy of JWKXS in the treatment of MCI by assessing the learning and memory abilities of SAMP8 mice. The mechanism was predicted using UPLC-Q-TOF/MS and systemic pharmacological analysis methods, and further validated *in vitro* and *in vivo*. Finally, the effect and mechanism of JWKXS were investigated in the MCI model of SAMP8 mice.

## 2. Materials and Methods

### 2.1. Preparation of Herbal Extracts

JWKXS was composed of *Cistanche deserticola* Y.C.Ma (30 g), *Ginseng quinquefolium* (L.) Alph.Wood (15 g), *Polygala tenuifolia* Willd (15 g), *Astragalus membranaceus* (Fisch.) Bunge (15 g), *Poria cocos* (Schw.) Wolf (15 g), *Ligusticum wallichii* Franch (15 g), and *Acorus tatarinowii* Schott (15 g). All herbal components were purchased from Beijing Tongrentang Nankai pharmacy Co., Ltd. (Tianjin, China).

JWKXS preparation was performed as previously reported [23]. In brief, the decoction pieces were mixed and boiled in eight volumes of double distilled water for 30 min and extracted third before filtering through gauze. The combined extracts were concentrated to 1.5 g/ml in a water bath.

### 2.2. Animals' Husbandry and Drug Administration

**Figure 1:**
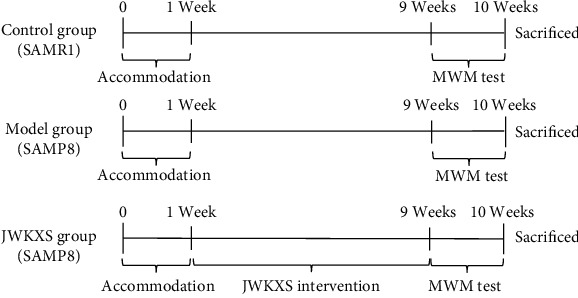
Flowchart of the experiment.

Four-month-old (20 ± 2 g, weight), 6-month-old (24 ± 2 g, weight), and 8-month-old (30 ± 2 g, weight) male SAMP8 and the corresponding age SAMR1 were provided by the Department of Medicine of Peking University School (Qualified No. SCXK 2016-0010, Beijing, China). The mice were housed at 24°C in cages with access to free food and water during the 12/12 hr light/dark cycle. Following a 7-day acclimatization period, the 4-month-old mice were randomly assigned to four groups with 15 mice in each group: the control (SAMR1) group, the model (SAMP8) group, the JWKXS-H (JWKXS-H+SAMP8) group, and the JWKXS-L (JWKXS-L + SAMP8) group. Mice in JWKXS-H (13 g/kg) and JWKXS-L (6.5 g/kg) group were administered with 13 and 6.5 g/kg JWKXS, while mice in control group and model group were treated with the same volume of double distilled water for 8 weeks (Figure 1). The study was approved by the Tianjin University of Traditional Chinese Medicine Animal Ethics Committee (TCM-LAEC-2020115).

### 2.3. Morris Water Maze Test

The spatial learning and memory ability of the mice were evaluated by Morris Water Maze test (MWM; Anhui Zhenghua biological instrument equipment Co., Ltd. China) [24]. Mice underwent a 6-day continuous training program conducted twice daily before devoting space exploration and navigation tests on Day 7. The escape latency, the times of crossing platform, and the residence time in the target quadrant were recorded.

### 2.4. Cerebral Blood Flow (CBF)

After the Morris water maze test, CBF was assessed using the Moor LDI2-HIR Laser Doppler (Moor Instruments). In brief, the mice were anesthetized by inhalation of isoflurane, and their heads were placed under the laser Doppler scanning imager. The whole brain was selected as the detection area for the base value of blood flow.

### 2.5. Histological Examinations

After fixing with formalin, the hippocampus was embedded with paraffin and was cut into 5-*μ*m-thick slices. In immunohistochemistry staining, after dewaxing, rehydration, and antigen retrieval, the hippocampus slices were treated with 3% H_2_O_2_. Next, the hippocampus slices were incubated with primary antibody rabbit anti-A*β*1–42 (1 : 200 dilutions, Sigma, USA) overnight at 4°C, and the secondary antibody (Affinity) was applied and incubated for 30 min. Finally, the slices were stained with DAB (Zhongshan Biotechnology, China) before being examined under a microscope. For the Nissl staining, the slices were stained with Cresyl violet and differentiated with Nissl liquid [25]. Hematoxylin and eosin (HE) staining was performed using HE. Congo red and hematoxylin were used in Congo red staining [26].

### 2.6. Western Blot

Total proteins were extracted from the hippocampus (*in vivo*) or BV2 microglial cells and PC12 cells (*in vitro*). Western blot experiments were performed as described by Zeng et al. [27]. Mouse anti-TLR4 (Santa Cruz, USA), rabbit anti-NLRP3 (ABCAM, UK), rabbit anti-IL-1*β* (ABCAM, UK), rabbit anti-cleaved-CASP1/anti-CASP1 (Wanleibio, China), rabbit anti-cleaved-CASP3/anti-CASP3 (Wanleibio, China), mouse anti-Fas (Santa Cruz, USA), rabbit anti-*β*-actin (Proteintech, USA), mouse antiapoptosis-associated speck-like protein containing a CARD (ASC) (Santa Cruz, USA), rabbit anti-IL-18 (Invitrogen, USA), and rabbit anti-GAPDH (Affinity, China), rabbit anti-IL-6 (Abclonal, China), rabbit anti-cleaved-CASP8/anti-CASP8, rabbit anti-cleaved-PARP/anti-PARP, rabbit anti-TNF-*α*, rabbit anti-Bcl2, rabbit anti-Bax, and rabbit anti-Bcl-xl (Cell Signaling Technology, USA) were used. Then, goat anti-rabbit IgG (Affinity, China) and horse anti-mouse IgG (Cell Signaling Technology, USA) were used as secondary antibodies.

### 2.7. Immunofluorescence

The frozen sections of the hippocampus were permeabilized in 0.3% Triton X-100 for 30 min at 37°C, then blocked by 10% goat serum for 30 min. Primary antibody ASC (1 : 50 dilution, Santa Cruz, USA) was incubated overnight at 4°C. Next, after 1 hr of FITC staining, DAPI counterstaining was applied for 5 min. Finally, hippocampus sections were visualized using Leica microsystem (Leica, Germany) and the fluorescence intensity was quantified using ImageJ.

### 2.8. Preparation of Drug-Containing Serum

The majority of conventional Chinese medicines are administered orally, rendering them unsuitable for *in vitro* experimentation. Consequently, the extraction of drug-containing serum becomes imperative for the purpose of conducting *in vitro* studies. The rats were randomly divided into JWKSX-H-containing serum group, JWKSX-L-containing serum group, and normal serum group. For the JWKSX-H-containing serum group and JWKSX-L-containing serum group, rats were given 13 and 6.5 g/kg JWKXS, respectively. Rats of the normal group were given the same volume of double distilled water for 7 days. On the 7th day, the blood samples of rats were collected from abdominal aorta, and serum was acquired via centrifugation, inactivation, and filtration.

### 2.9. Cell Culture

BV2 microglia cells were purchased by Wuhan Pronosa Life Science and Technology Co., Ltd. The PC12 cell line (rat adrenal pheochromocytoma) was purchased from the Institute of Basic Medicine, Chinese Academy of Medical Sciences. The BV2 microglial cells and PC12 cells were cultured in DMEM and RPMI-1640 medium, respectively. Both of these cells were randomly into four groups: control group (addition of 10% FBS), A*β*1–42 group (addition of 2.5 *μ*M A*β*1–42), JWKXS-H group (addition of 10% JWKSX-H-medicated serum, and 2.5 *μ*M A*β*1–42), and JWKXS-L group (addition of 10% JWKSX-L-medicated serum, and 2.5 *μ*M A*β*1–42). To obtain differentiated PC12 cells, we changed the medium every other day for 5 days to a serum-free medium containing NGF (50 ng/ml). Then the cells were cultured in RPMI-1640 medium. All the subsequent experiments in this study were performed using these differentiated PC12 cells.

### 2.10. Preparation of A*β*1–42

The initial solution of 10 mg A*β*1–42 peptide (Sigma, USA) was dissolved in 2.2 ml hexafluoroisopropanol (HFIP), aliquoted, and stored on an HFIP film at 20°C. After HFIP was evaporated, the aliquoted peptide was resuspended in 10 *μ*l DMSO (Solarbio, China) to a concentration of 10 mM and then diluted in 10 *μ*l DMEM/F12 serum-free medium to a concentration of 5 mM. The peptide used for preparing the oligomer was incubated at 4°C for 24 hr, centrifuged at 14,000 *g* 4°C for 10 min, and the oligomer was found in the supernatant. DMEM/F12 serum-free medium was continuously diluted to different concentrations.

### 2.11. Statistics

Statistical data were shown as means ± SD. SPSS 22.0 software was used to analyze data while GraphPad Prism (version 8.0) was used to draw graphs. One-way ANOVA with Newman–Keuls, a multiple comparisons test, was applied to the normal distribution and homogeneous variance, while Dunnett' T3 test was used for nonnormal distribution. Two-way analysis of variance with repeated measures followed was conducted to assess escape latency in the Morris water maze test during the training period. *P* < 0.05 indicates statistical significance.

## 3. Result

### 3.1. Selection of 4-Month-Old SAMP8 Mice for MCI

**Figure 2:**
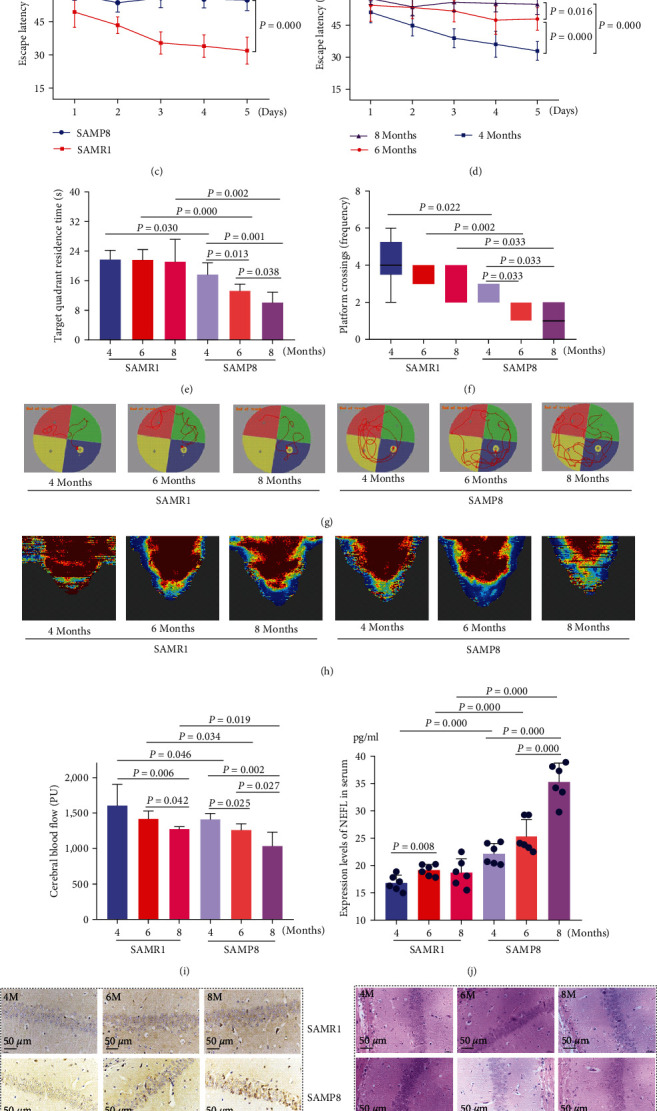
Selection of 4-month-old SAMP8 mice for MCI. (a–c) The escape latency of 4-, 6-, and 8-month-old SARM1 and SAMP8 mice. (d) The escape latency of SAMP8 mice at 4-, 6-, and 8-month-old. (e) Target quadrant residence time, (f) platform crossing times and (g) swimming trajectories of SAMR1 and SAMP8 mice at 4-, 6-, and 8-month-old were measured in the MWM test. (h, i) Cerebral blood flow was measured using the Moor LDI2-HIR Laser Doppler. (j) The content of serum NEFL. Representative images of the hippocampus from (k) immunohistochemical staining of A*β*, (l) HE staining, and (m) Congo red staining. The magnification was 400x (scale bar = 50 *μ*m). Data are expressed as mean ± SD and analyzed with one-way ANOVA; two-way ANOVA only was performed in the escape latency during the Morris water maze test.

First, the MWM, CBF, NEFL content, and morphological detection were used for screening suitable animal models of MCI. MWM showed that when compared with the age-matched SAMR1 group, the escape latency prolonged (Figure 2(a)–2(c)), target quadrant residence time (Figure 2(e)), and platform crossing times (Figure 2(f)) shortened and the mice could not find the hidden platform in water more smoothly (Figure 2(g)) in the 4-, 6-, 8-month-old SAMP8 mice. As the age increased, the escape latency was longer (Figure 2(d)), the target quadrant residence time and platform crossing times were less, and the mice were less likely to find the hidden platform. These changes suggested that the learning and memory ability of SAMP8 mice decreased gradually as their age increased. Besides, the result of CBF was consistent with those of the MWM (Figures 2(h) and 2(i)). Finally, morphological examination showed that the hippocampal structure and cell morphology of SAMP8 mice changed abnormally (Figure 2(l)). These changes were accompanied by different degrees of A*β* deposition (Figure 2(k)) and the formation of punctate amyloid plaques (Figure 2(m)). Additionally, the content of serum NEFL increased (Figure 2(j)) as the age of mice increased, and these injuries aggravated gradually. Therefore, 4-month-old SAMP8 mice were used as a follow-up model group.

### 3.2. JWKXS-Treatment Ameliorated MCI

**Figure 3:**
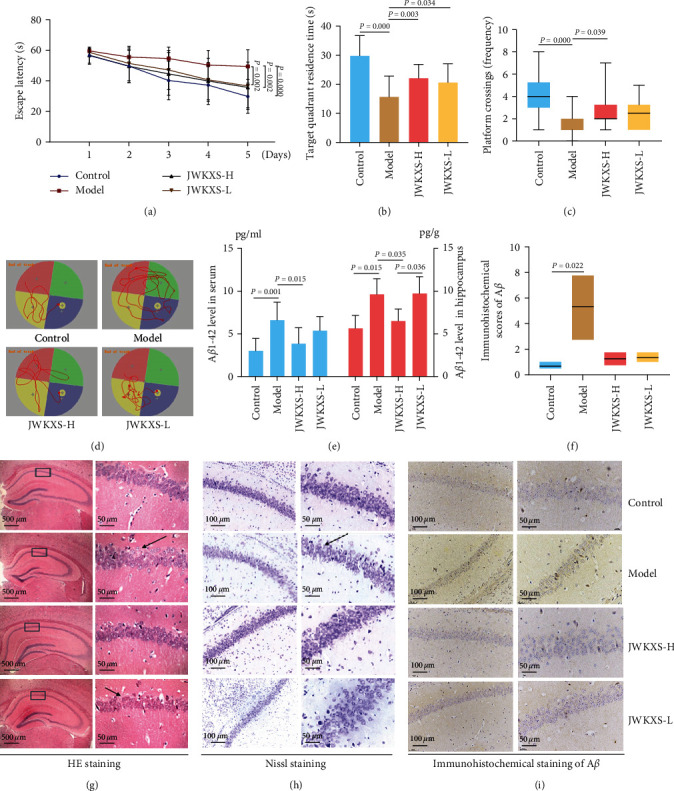
JWKXS treatment ameliorated MCI. (a) Escape latency, (b) target quadrant residence time, (c) platform crossing times, and (d) swimming trajectories were measured through the MWM test. (e) Quantitative analysis of the content of A*β* in serum and hippocampus. Representative images of hippocampus showing the (g) HE staining, (h) Nissl staining, and (i) immunohistochemical examination of A*β*, and (f) Image-Pro Plus software was used to analyze the immunohistochemistry results. The original magnification was 40x (scale bar = 500 *μ*m), medium-power magnification was 200x (scale bar = 100 *μ*m), while the high magnification was 400x (scale bar = 50 *μ*m). Data are expressed as mean ± SD and analyzed with one-way ANOVA; two-way ANOVA only was performed in the escape latency during the Morris water maze test.

To investigate the functional effects of JWKXS on AD with MCI, the ability of learning and memory was detected by MWM. The neurons in the hippocampal CA1 region loosely arranged, and the Nissl bodies in the cytoplasm were fewer in the model group than those in the control group. Moreover, the content of A*β*1–42 in serum and hippocampal tissue increased, and a small amount of A*β* deposition was found in the hippocampal tissue in the model group. The JWKXS intervention shortened the escape latency of SAMP8 mice (Figure 3(a)), increased target quadrant residence time (Figure 3(b)), and the platform crossing times (Figure 3(c)), thus enabling the mice to find the hidden platform in water more smoothly (Figure 3(d)). These changes revealed that JWKXS can improve the learning and memory ability of SAMP8 mice. Additionally, JWKXS treatment decreased the content of A*β*1–42 in serum and hippocampal tissue (Figure 3(e)). Still, it did not affect the deposition of A*β* detected by immunohistochemical staining (Figures 3(f) and 3(i)) in hippocampal tissue. Finally, after JWKXS intervention, the neurons in the hippocampal CA1 area were arranged more regularly, the number of cells increased (Figure 3(g)), and the content of Nissl bodies in the cytoplasm increased (Figure 3(h)). These findings suggested that JWKXS contributed to the overall improvement of MCI in SAMP8 mice.

### 3.3. JWKXS-Improved MCI by Regulating Inflammation and Apoptosis Signaling Pathways

**Figure 4:**
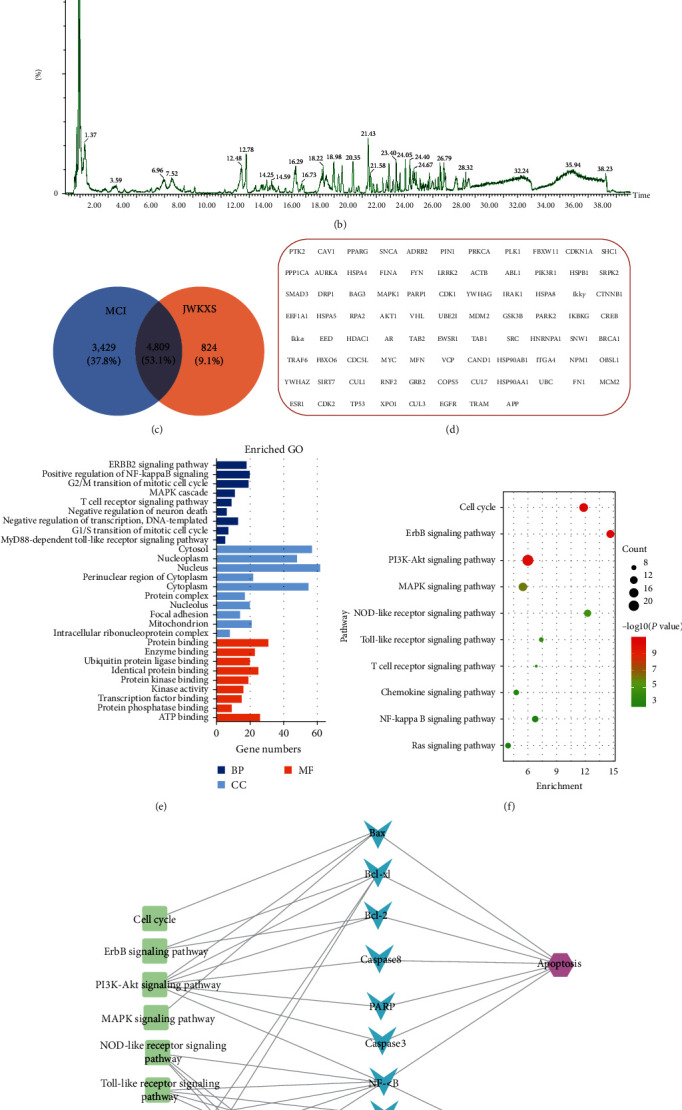
JWKXS improved MCI by regulating inflammation and apoptosis signaling pathways. (a) Total ion current BPI chromatogram in positive mode and (b) negative mode of JWKXS extract. (c) The intersection of MCI and JWKXS related targets was used to obtain, (d) 85 target genes, which were enriched by (e) GO and (f) KEGG, and finally (g) the predicted targets of JWKXS in the treatment of MCI identified through pathway analysis.

There were 91 compounds (*Supplementary 1*) that were identified by UPLC-Q-TOF/MS analysis of JWKXS extract (Figures 4(a) and 4(b)). Based on the compounds identified in the JWKXS extract, 5,633 compound targets were achieved from TCMSP and Swiss Target Prediction databases. In addition, 8,238 MCI-related targets were obtained from OMIM, TTD, and Drugbank databases. Finally, interaction networks between the drug-targets and disease-targets were constructed, and the results were intersected to obtain 4,809 genes (Figure 4(c)). In this study, degree >143 and betweenness centrality >0.0021 were selected as the screening conditions for the core nodes, and 85 candidate targets of JWKXS for treating MCI were finally obtained (Figure 4(d)). The DAVID 6.8 online analysis tool was used for performing GO (Figure 4(e)) and KEGG enrichment analysis (Figure 4(f)) on the 85 screened core targets. The tops 10 significant enrichment analysis results were obtained by combining the “count value” and *P* ≤ 0.05. The effects of the core targets enriched in the key pathways of the network were analyzed by the outcome effect-based pathway analysis. The effector targets of JWKXS that improved MCI were NF-*κ*B, TLR4, TNF-*α*, IL-6, NLRP3, IL-1*β*, IL-18, Bax, Bcl2, Bcl-xl, CASP8, PARP, and CASP3. Furthermore, we explored the pathways associated with the above effector targets and found the regulatory indicators were mainly related to the TLR4/NF-*κ*B pathway, NLRP3 inflammasome pathway, and the classic intrinsic and extrinsic signaling pathways of apoptosis. The relation to these pathways suggested that apoptosis and inflammation were the key effects of JWKXS for improving MCI (Figure 4(g)).

### 3.4. JWKXS-Inhibited Inflammation through the TLR4/NF-*κ*B and NLRP3 Inflammasome Pathways

**Figure 5:**
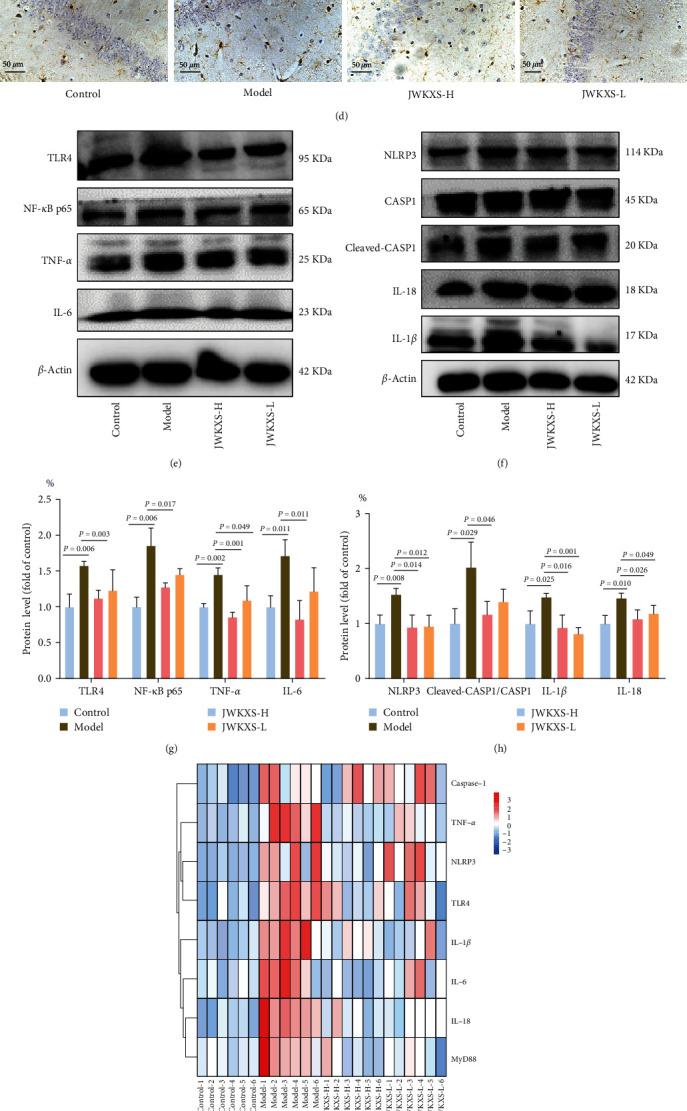
JWKXS inhibited inflammation through the TLR4/NF-*κ*B and NLRP3 inflammasome pathways. (a, b) TNF-*α*, IL-1*β*, and IL-6 levels in the serum and hippocampus. (c, d) Representative immunohistochemical-staining images and quantitative analysis of Iba-1 in the hippocampus. (e–i) The mRNA and protein expression levels of TLR4, NF-*κ*B p65, TNF-*α*, IL-6, NLRP3, IL-1*β*, IL-18, and cleaved-CASP1/CASP1 in the TLR4/NF-*κ*B and NLRP3 inflammasome pathways in the hippocampus. GAPDH and *β*-actin served as the internal control. (j) Fluorescence images and quantitative analysis of ASC in the hippocampus of mice. The original magnification of immunohistochemical-staining images was 200x (scale bar = 100 *μ*m), while the high magnification was 400x (scale bar = 50 *μ*m). The magnification of ASC fluorescence images was 400x (scale bar = 50 *μ*m). Data are presented as mean ± SD.

Based on the work ahead of us, the key effect targets in the inflammation signaling pathway were constructed. As shown in Figures 5(a) and 5(b), TNF-*α*, IL-1*β*, and IL-6 levels in serum and hippocampus were significantly higher in the model group than in the control group. Similarly, the number of Iba-1 positive cells increased in the CA1 region of the hippocampal, with cells showing dendritic morphology (Figures 5(c) and 5(d)). JWKXS significantly reduced the levels of the above inflammatory factors and the number of Iba-1 positive cells in the CA1 region of the hippocampal. Then, the qRT-PCR and western blot were used to detect the mRNA and protein expressions. It was found that the TLR4, NF-*κ*B p65, TNF-*α*, and IL-6 in the TLR4/NF-*κ*B pathways and NLRP3, IL-1*β*, IL-18, and cleaved-CASP1/CASP1 in NLRP3 inflammasome pathways were significantly increased in the hippocampus of the model group, which were reversed by the JWKXS treatment (Figure 5(e)–5(i) and *Supplementary 1*). Furthermore, ASC oligomerization, a key step in NLRP3 inflammasome activation, increased in the hippocampal CA1 region of model group, which was reduced by JWKXS treatment (Figure 5(j)).

### 3.5. JWKXS-Ameliorated MCI via the Intrinsic and Extrinsic Apoptotic Signaling Pathways

**Figure 6:**
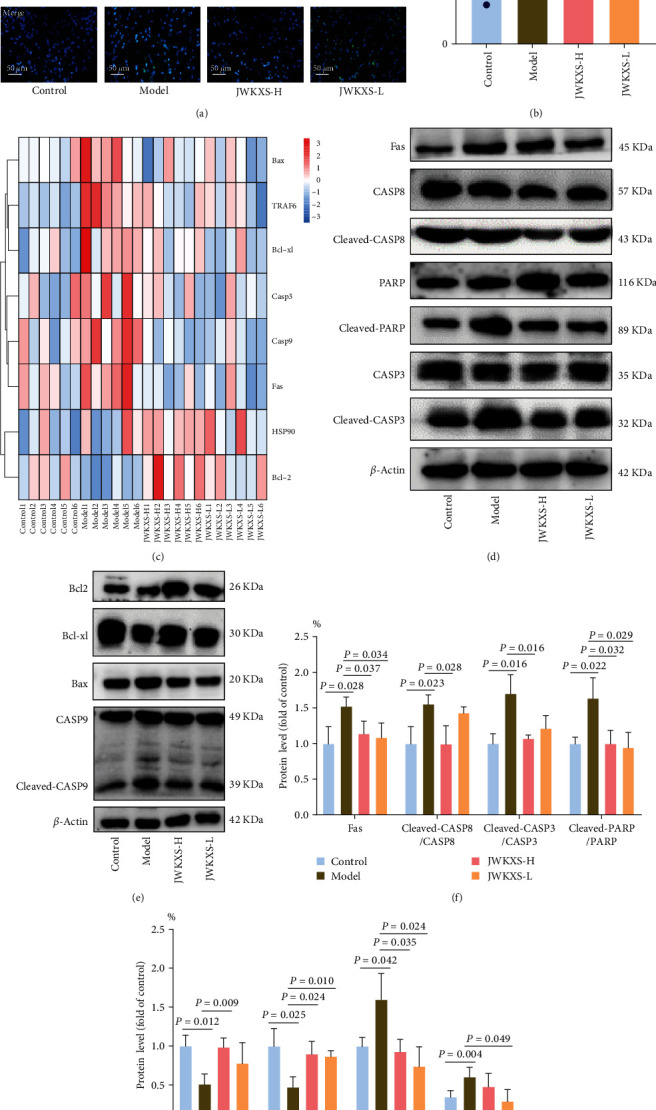
JWKXS ameliorated MCI via the intrinsic and extrinsic apoptotic signaling pathways. (a, b) TUNEL stained images in hippocampus and the apoptotic cell index. (c) The transcription levels of apoptosis related genes. (d–g) The extrinsic and intrinsic apoptosis related proteins. GAPDH and *β*-actin served as the internal control. The magnification of TUNEL stained images was 400× (scale bar = 50 *μ*m). Data are presented as mean ± SD.

To study whether the apoptosis injury was involved in MCI, the apoptosis index was performed using TUNEL staining. JWKXS significantly reduced the number of TUNEL-positive cells in the brain of SAMP8 mice (Figures 6(a) and 6(b)). Then, combined with the results of network pharmacology, the expressions of key effect targets in the apoptosis signaling pathway were conducted. The mRNA expression level of antiapoptotic factors Bcl2 and Bcl-xl were downregulated, while the proapoptotic factors Bax, Fas, CASP9, and CASP3 were upregulated but restored with JWKXS treatment, as shown in Figure 6(c) and *Supplementary 1*. The expression levels of extrinsic apoptosis-related proteins, namely Fas, cleaved-CASP8/CASP8, cleaved-CASP3/CASP3, and cleaved PARP/PARP were found to be significantly elevated in the model group. However, this increase was reversed upon JWKXS administration (Figures 6(d) and 6(f)). Moreover, the JWKXS group exhibited significantly elevated protein expression levels of Bcl2 and Bcl-xl, while demonstrating decreased expression levels of Bax and cleaved-CASP9/CASP9 compared to the model group (Figures 6(e) and 6(g)). These results indicated that JWKXS-ameliorated MCI involving the intrinsic apoptosis pathway. Therefore, JWKXS regulated the expression levels of key target genes in intrinsic and extrinsic apoptotic signaling pathways, reducing neuronal apoptosis, which are the main mechanism underlying its neuroprotective effect.

### 3.6. JWKXS Treatment Inhibited A*β*1–42 Induced BV2 Microglial Cells Inflammation

**Figure 7:**
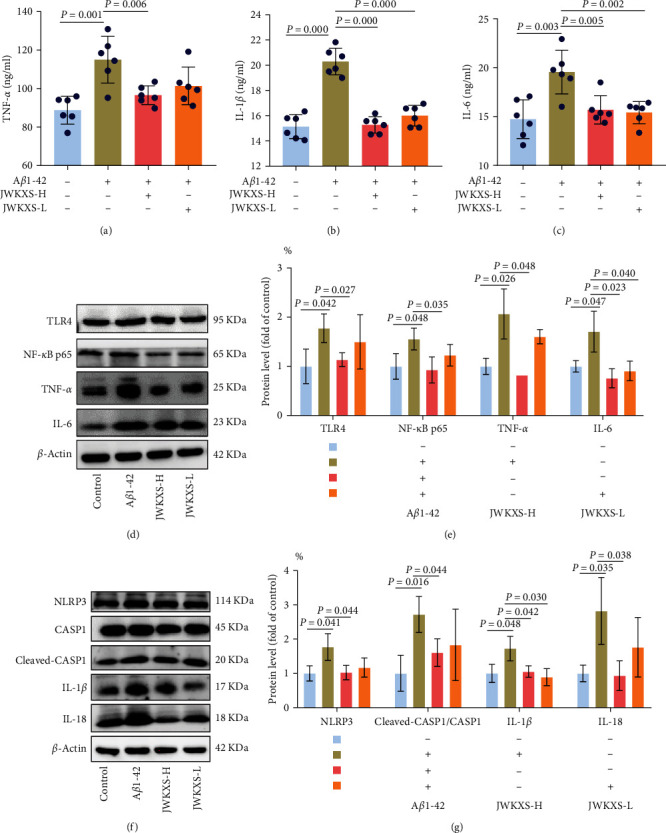
JWKXS treatment inhibited A*β*1–42 induced BV2 microglial cells inflammation. (a–c) The contents of inflammatory cytokines TNF-*α*, IL-1*β*, and IL-6 in the cell culture supernatants. (d, e) The protein expression levels of TLR4, NF-*κ*B P65, TNF-*α*, and IL-6. (f, g) The protein expressions of NLRP3, CASP1, cleaved CASP1/CASP1, IL-1*β*, and IL-18 in A*β*1–42-induced BV2 microglial cells. GAPDH and *β*-actin served as the internal control. Data are presented as mean ± SD.

To further verify *in vivo* results, we used different A*β*1–42 concentrations to induce an inflammatory response. It was found that 2.5-*μ*M A*β*1–42 can successfully replicate the BV2 microglial cells activation model (*Supplementary 1*). Besides, CCK8 assay showed that JWKXS was nontoxic (*Supplementary 1*). The levels of inflammatory cytokines TNF-*α*, IL-1*β*, and IL-6 increased in the cell culture supernatants in the A*β*1–42 group, which were markedly reduced in the JWKXS group (Figure 7(a)–7(c)). The protein expression levels of TLR4, NF-*κ*B P65, TNF-*α*, IL-6, NLRP3, cleaved-CASP1/CASP1, IL-1*β*, and IL-18 in the TLR4/NF-*κ*B and NLRP3 inflammasome pathways were upregulated in A*β*1–42 group, and JWKXS downregulated the expression levels of these proteins (Figure 7(d)–7(g)). Thus, JWKXS can mainly inhibit inflammation by inhibiting TLR4/NF-*κ*B and NLRP3 inflammasome pathways in A*β*1–42 induced BV2 microglial cells.

### 3.7. JWKXS Treatment Prevented A*β*1–42 Induced PC12 Cells Apoptosis

**Figure 8:**
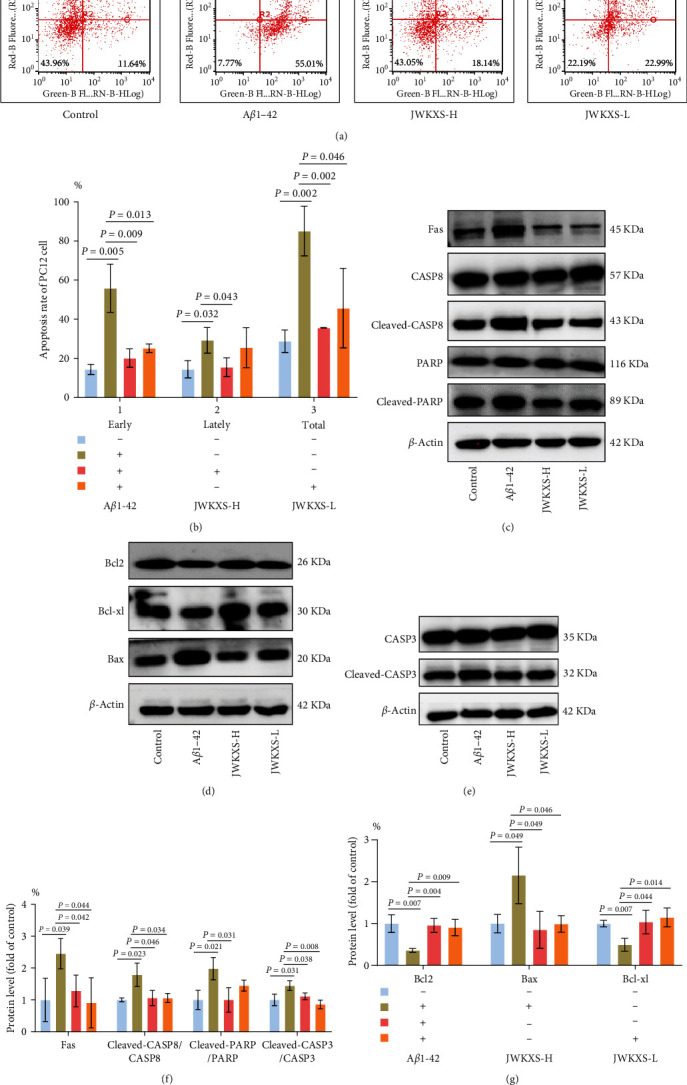
JWKXS treatment prevented A*β*1–42 induced PC12 cells apoptosis. (a) Cell apoptosis analysis by flow cytometry. (b) Apoptotic rate (%) of early and late-stage apoptotic cells. (c–g) The protein expression levels of apoptotic factors Fas, cleaved-CASP8/CASP8, cleaved-PARP/PARP, cleaved-CASP3/CASP3, and Bax, Bcl2 and Bcl-xl. GAPDH and *β*-actin served as the internal control. Data are presented as mean ± SD. FITC: fluorescein isothiocyanate; PI: propidium iodide.

The apoptosis model was successfully replicated by PC12 cells treated with 2.5 *μ*M A*β*1–42 at the same concentration in BV2 microglial cells. The rates of early and late neuronal apoptosis significantly increased in the A*β*1–42 group, whereas JWKXS treatment effectively reduced the rate of neuronal apoptosis (Figures 8(a) and 8(b)). In the A*β*1–42 group, the protein expression levels of apoptotic factors Fas, cleaved-CASP8/CASP8, cleaved-PARP/PARP, cleaved-CASP3/CASP3, and Bax were all upregulated. In contrast, the expression levels of antiapoptotic factors Bcl2 and Bcl-xl were significantly downregulated, and JWKXS administration reversed these results (Figure 8(c)–8(g)).

## 4. Discussion

As a high-risk state for developing AD, MCI is also characterized by histopathological changes of the brain, impaired cholinergic system, A*β* deposition, cerebrovascular changes, and age-related deterioration of learning and memory but does not affect daily life [7]. Previous studies suggested that “middle-aged” SAMP8 mice were a suitable model for basic MCI research. Moreover, studies revealed that 5-month-old SAMP8 mice were equivalent to the “middle-aged” population, and the main manifestations were cognitive and neurobiological changes due to A*β* deposition [28]. Besides, Kang et al. [28] reported that the 3-month-old SAMP8 and SAMR1 mice showed similar their learning and memory abilities. Building on these findings, SAMP8 mice were selected at three different ages: 4-, 6-, and 8-month-old, to conduct a study where stages of MCI were determined by behavior observation and brain morphology. Our results showed that 4-month-old SAMP8 mice had a fluctuant trend of CBF, elevated-serum NEEL levels, and early age-related A*β* deposition in hippocampal tissue, leading to changes in cognitive and cerebral histopathology. Based on these findings, MCI was present in 4-month-old SAMP8 mice, which is a reliable time point for pharmacological intervention for preventing the progression of MCI to AD.

Since MCI is characterized by cognitive decline, including the ability to learn and remember, delaying cognitive decline is an important criterion for assessing drug efficacy [29]. In this study, MWM experiment results showed that JWKXS dramatically lowered the escape latency, increased the times of crossing platform, and prolonged the residence time in the target quadrant, thereby delaying MCI progression. In another study by Zhu et al. [21], JWKXS, originating from two famous formulae, KXS and Sheng-Mai-San, was used to treat A*β*1–42 induced cognitive deficit mice. This treatment improved cognitive deficit mainly by lowering A*β* levels in the hippocampus, inhibiting the oxidation damage, and raising powerful neurotrophic activity. The above results have confirmed that JWKXS, based on KXS, could play the role of amplification. Altogether, JWKXS was a novel therapeutic agent for treating MCI. The characteristics of multiple components and targets make the therapeutic mechanisms of JWKXS extremely complex. In this study, “drug-targets” and “disease-targets” network were constructed via network pharmacology to obtain the key targets of JWKXS for improving MCI. Finally, inflammation and apoptosis signaling pathways were found to be the key effects of JWKXS on MCI treatment, as per the pathological mechanism of MCI [30, 31].

Neuroinflammation is involved in the pathogenesis of MCI [32, 33]. Early autopsy results concurred with our finding that many activated microglia aggregates were present in the brain of AD (especially around the hippocampus) with cognitive impairment [34]. This study found that JWKXS intervention significantly reduced microglia activation in the hippocampus of SAMP8 mice. These results suggested that an anti-inflammation effect of JWKXS targets microglia. The inflammation effector genes in PPI networks were TLR4, NF-*κ*B, NLRP3, IL-1*β*, IL-18, TNF-*α*, and IL-6 of the TLR4/NF-*κ*B/NLRP3 inflammasome pathway which was a key signaling mediator that activated inflammation microglia triggered by A*β* [35]. NLRP3 Inflammasome is responsible for producing proinflammatory cytokines such as IL-1*β* and IL-18, associated with the pathogenesis of MCI [36]. Study has shown that NLRP3 deficiency significantly attenuates the neuroinflammatory response and ameliorates AD-like changes in APP/PS1 mice [37]. Moreover, as a classical inflammatory signaling pathway, activating TLR4/NF-*κ*B signaling pathway is a fundamental step in forming NLRP3 inflammasome [38–40]. Additionally, it has been established that TLR4 and NF-*κ*B p65 have participated in the microglia M1 polarization to promote the secretion of proinflammatory cytokines [41, 42]. Our findings demonstrated that the mRNA and protein expression levels of TLR4, NF-*κ*B p65, NLRP3, IL-1*β*, IL-18, and cleaved-CASP1/CASP1 were increased in the hippocampus of SAMP8 mice, suggesting that the TLR4/NF-*κ*B pathway and NLRP3 inflammasome pathway were activated. JWKXS downregulated the expression levels of genes related to the inflammation above, inhibited NLRP3 inflammasome activation, and reduced the production and release of inflammatory factors, resulting in anti-neuroinflammation effects of MCI SAMP8 mice. Furthermore, we used A*β*1–42 induced BV2 microglial cells activation model to confirm the similar results *in vivo*.

Precisely, the activation of microglia might cause an imbalance in the homeostasis of brain [43]. Neuronal apoptosis is believed to occur during the development of MCI [29]. In the present study, the apoptosis effector genes in PPI networks were Bcl2, Bcl-xl, Bax, CASP8, PARP, and CASP3. Basic science research [44] showed that the neuronal apoptosis was significantly alleviated mice by KXS treatment in the brain of SAMP8. In the intrinsic apoptosis pathway, the proapoptotic Bcl2 family members, such as Bax, stimulate CASP9 activation by permeabilizing mitochondrial membranes and releasing cytochrome C. [45]. KXS inhibited Bax expression and increased Bcl2 expression, which improved the scopolamine-induced cognitive dysfunction of Kunming mice [9]. However, more studies are needed to elucidate the effects of JWKXS on the injury of neurons in MCI. Therefore, we investigated the impact of JWKXS on neuroprotective functions and possible mechanisms. Our results demonstrated that JWKXS decreased the expression level of proapoptotic factors Bax and CASP9, and increased the expression levels of antiapoptotic factors Bcl2, Bcl-xl in the intrinsic apoptosis pathway in MCI SAMP8 mice and A*β*1–42 induced PC12 cells. This finding demonstrated that JWKXS treatment inhibited the intrinsic apoptosis pathway in a specific way.

In the extrinsic apoptosis pathway, elevated levels of Fas, a cell-surface receptor implicated in apoptosis initiation, have been reported in the brain of AD patients [46]. When death receptor like Fas on the cell surface is activated, CASP8, which belongs to a family of cysteine aspartate-specific proteases, can initiate extrinsic apoptosis [47]. Besides, CASP8 was reported to mediate A*β*-induced neuronal apoptosis *in vivo* and extensive evidence suggested that CASP3 was involved in AD [48]. Our results indicated that neuronal apoptosis in MCI SAMP8 mice was regulated by the orderly activation of Fas, CASP8, and CASP3. PARP was the cleavage substrate of CASP3 in the extrinsic apoptosis pathway. Our studies showed that JWKXS inhibited the protein expression of death receptor Fas, CASP8, apoptosis effector protein cleaved-CASP3, and cleaved-PARP in MCI SAMP8 mice and A*β*1–42 induced PC12 cells. Overall, the results suggested that JWKXS functions as a neuroprotective agent by regulating the expression of key target genes in the intrinsic and extrinsic apoptosis pathways.

## 5. Conclusion

The present study demonstrated that JWKXS treatment could improve learning and memory ability and exerted a neural protective effect against MCI via anti-inflammation and antiapoptosis. The ability of JWKXS to treat MCI effectively suggested that JWKXS needed to be evaluated in the clinical studies for the prevention and treatment of AD.

## Data Availability

The data used to support the findings of this study are available from the corresponding author upon request.

## Abbreviations

ASC: Apoptosis-associated speck-like protein containing a CARD
AD: Alzheimer's disease
A*β*: Age-related amyloid-*β*
DL: Druglikeness
HFIP: Hexafluoroisopropanol
Iba-1: Ionized calcium-binding adapter molecule-1
JWKXS: Jia-Wei-Kai-Xin-San
KXS: Kai-Xin-San
MCI: Mild cognitive impairment
MWM: Morris water maze
NGF: Nerve growth factor
OD: Optical density
OB: Oral bioavailability
OMIM: Online mendelian inheritance in man
PI: Propidium iodide
SAMP8: The senescence-accelerated mouse prone 8
SAMR1: Senescence resistant 1
TCM: Traditional chinese medicine
TCMSP: TCM systems pharmacology database and analysis platform
TTD: Therapeutic target database
TUNEL: Terminal deoxynucleotidyl transferase-mediated dUTP nick-end labeling.

## Supplementary Materials

Supplementary 1 Table S1: component analysis of JWKXS extract; Figure S1: (a, b) transcription levels of genes involved in inflammation and apoptosis. (c, g) Cellular viability for BV2 microglial cells were treated or not treated with JWKXS. (d–f) mRNA expression of inflammatory cytokines TNF-*α*, IL-6, and IL-1*β* in BV2 microglial cells intervened with different concentrations of A*β*1–42.

Supplementary 2 The sampling order of the blots is as follows: Control/Model/JWKXS-H/JWKXS-L/BYHWD. In addition to JWKXS, we actually provide BYHWD (Buyang Huanwu Decoction) to explore its mechanism. Due to some of its poor effects and unrelated to this article, it is not included. Original western blots were repeated three times.
