# Supplementary material for: Jia-Wei-Kai-Xin-San Treatment Alleviated Mild Cognitive Impairment through Anti-Inflammatory and Antiapoptotic Mechanisms in SAMP8 Mice
Source: Mediators Inflamm. 2023 Nov 2;2023:7807302. doi: 10.1155/2023/7807302 (PMC10635749; doi:10.1155/2023/7807302)
Supplement: Supplementary 1 — Table S1: component analysis of JWKXS extract; Figure S1: (a, b) transcription levels of genes involved in inflammation and apoptosis. (c, g) Cellular viability for BV2 microglial cells were treated or not treated with JWKXS. (d–f) mRNA expression of inflammatory cytokines TNF-α, IL-6, and IL-1β in BV2 microglial cells intervened with different concentrations of Aβ1–42. [file 7807302.f1.pdf]

Figure 4

	Repeat1	Repeat2	Repeat3
NLRP3	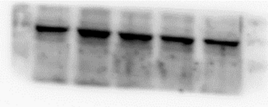	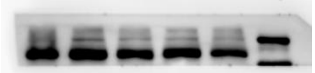	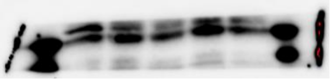
Casp-1/ Cleaved-Casp-1	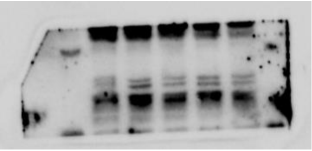	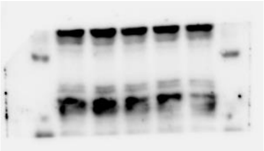	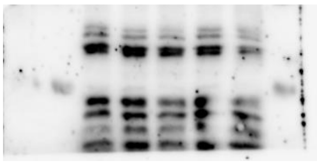
IL-18	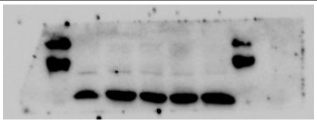	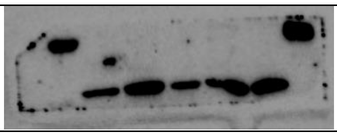	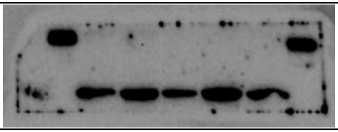
IL-1β	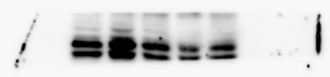	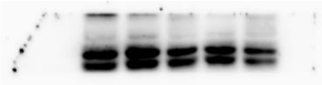	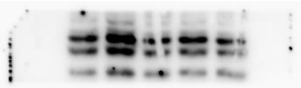
TLR4	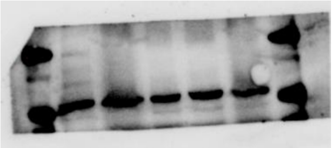	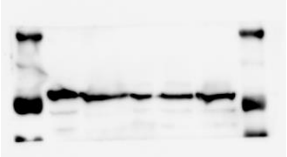	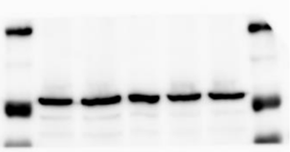
NF-κB	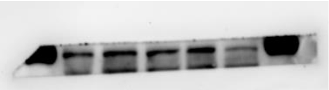	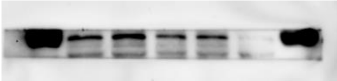	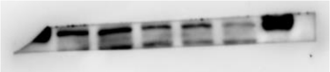
TNF-α	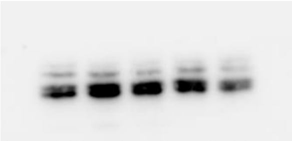	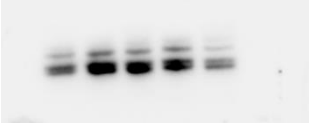	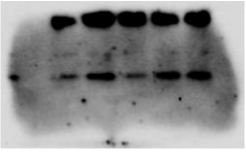
IL-6	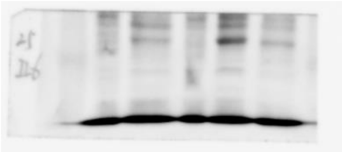	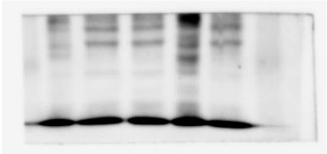	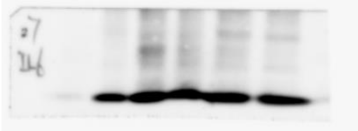

Figure 5

	Repeat1	Repeat2	Repeat3
**FAS**	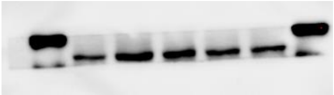	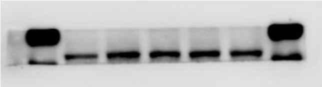	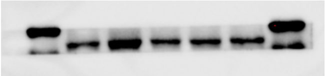
**Casp-8/**	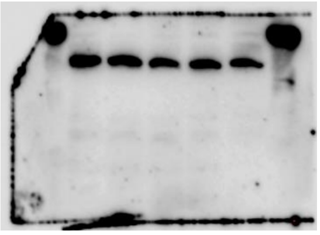	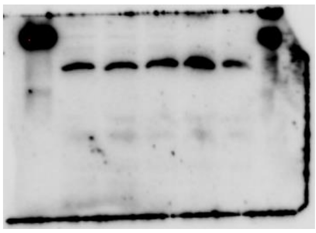	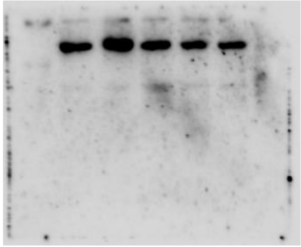
**Cleaved-Casp-8**	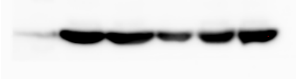	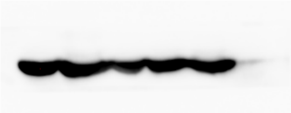	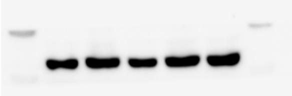
**PARP**	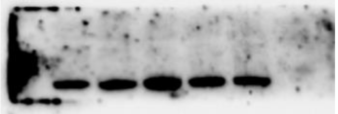	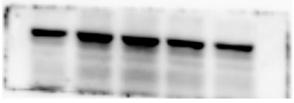	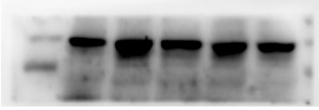
**Cleaved-PARP**	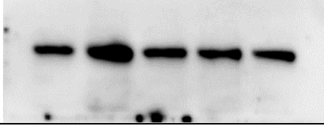	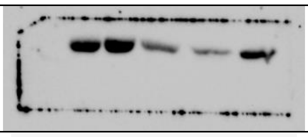	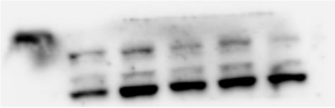
**Casp-3**	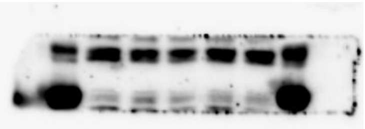	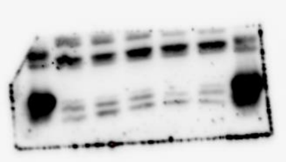	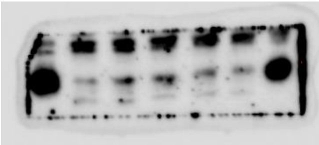
**Cleaved-Casp-3**	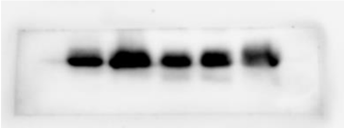	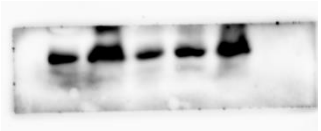	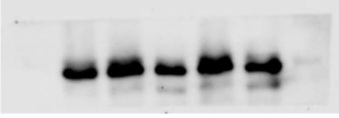
**Bcl-2**	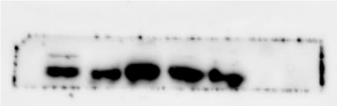	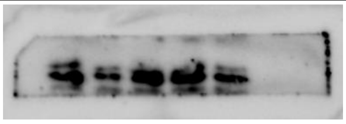	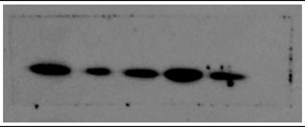
**Bcl-xl**	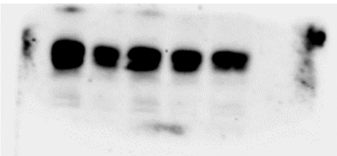	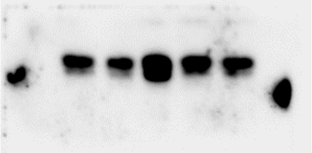	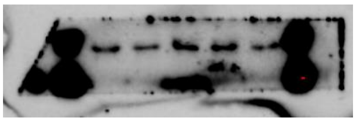
**Bax**	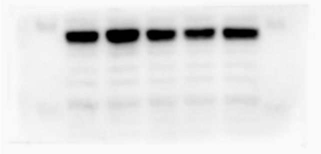	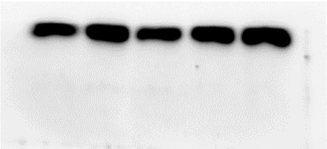	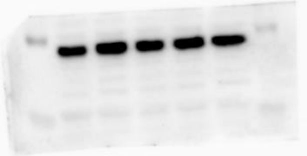

**Casp-9 Cleaved-Casp-9**	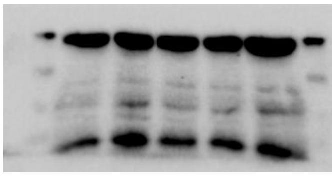	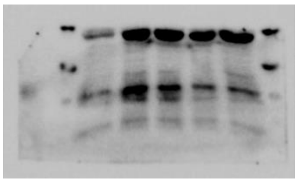	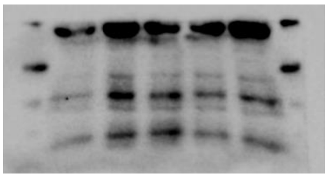

Figure 6

	Repeat1	Repeat2	Repeat3
**NLRP3**	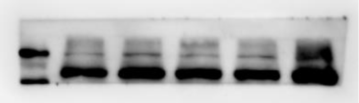	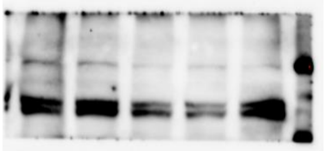	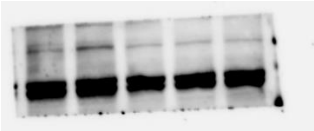
**Casp-1**	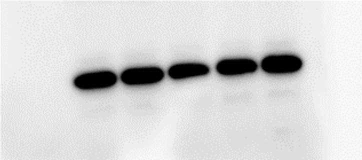	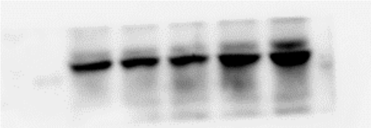	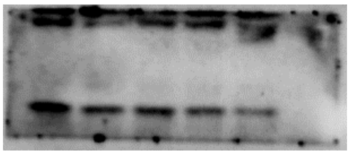
**Cleaved-Casp-1**	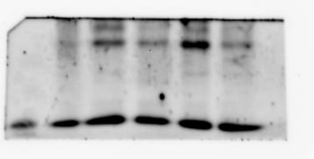	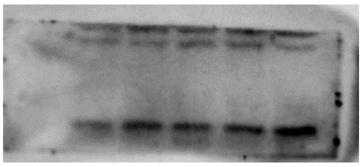	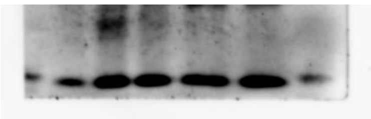
**IL-18**	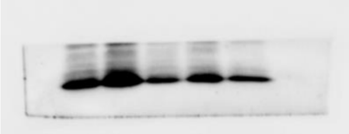	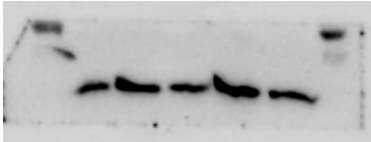	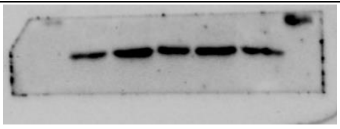
**IL-1β**	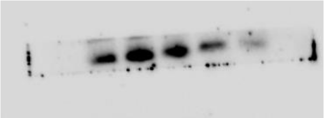	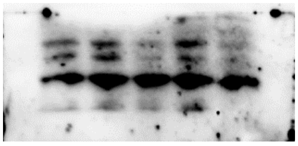	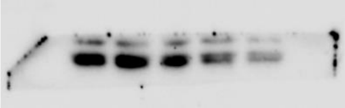
**TLR4**	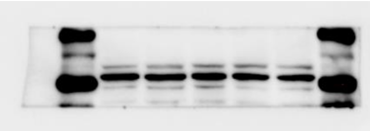	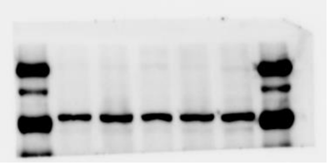	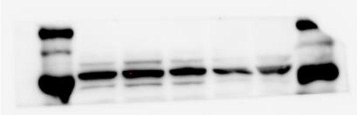
**NF-κB**	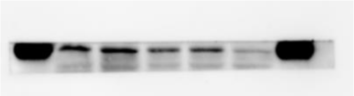	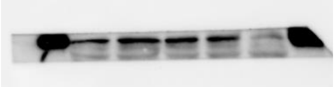	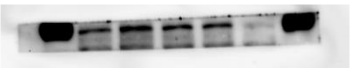
**TNF-α**	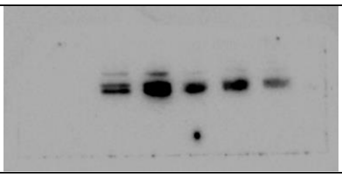	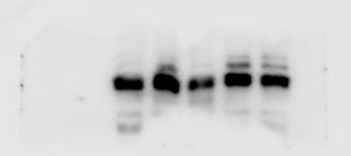	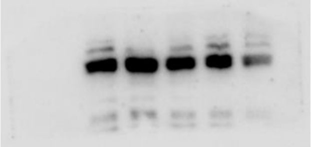
**IL-6**	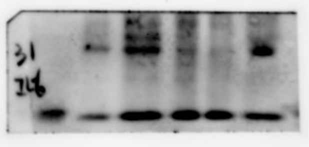	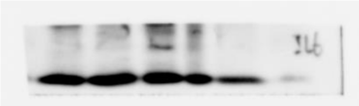	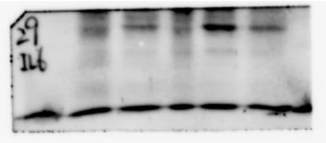

Figure 7

	Repeat1	Repeat2	Repeat3
**FAS**	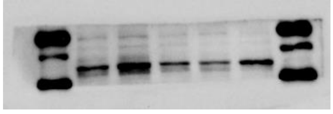	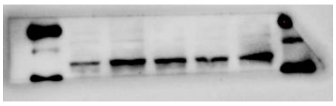	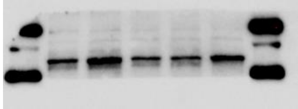
**Casp-8**	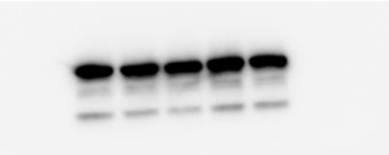	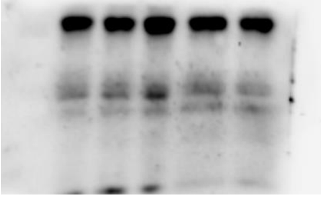	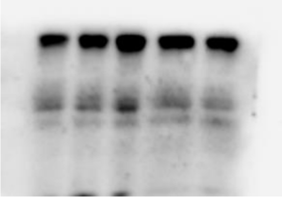
**Cleaved-Casp-8**	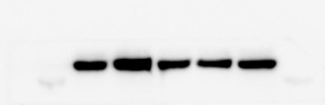	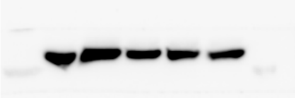	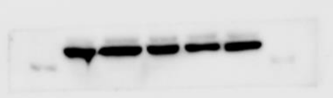
**PARP**	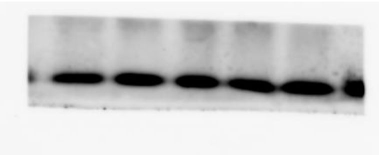	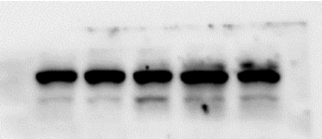	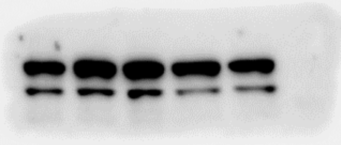
**Cleaved-PARP**	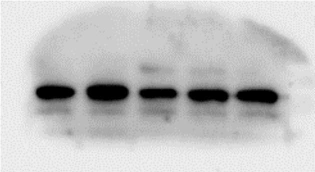	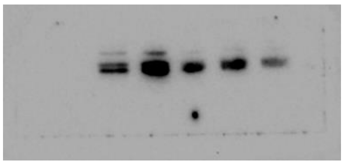	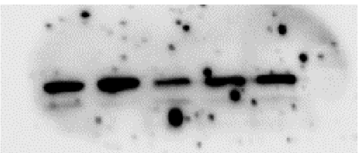
**Casp-3**	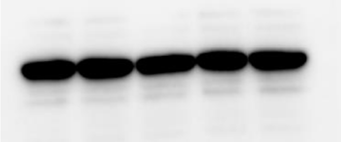	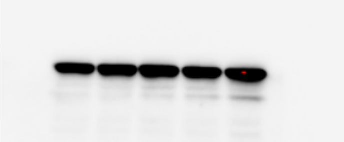	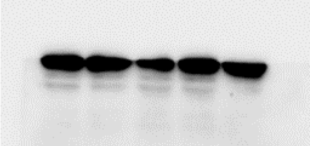
**Cleaved-Casp-3**	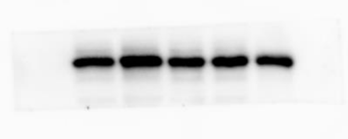	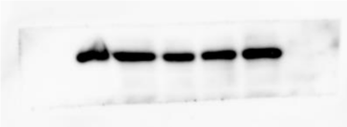	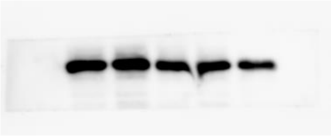
**Bcl-2**	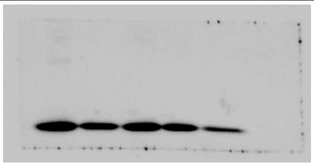	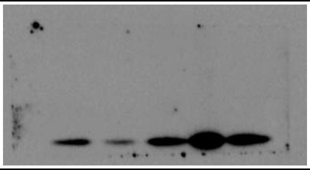	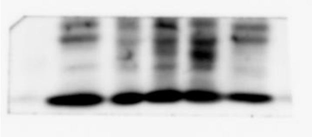
**Bcl-xl**	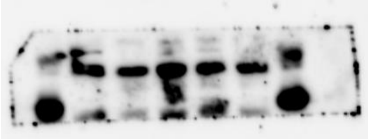	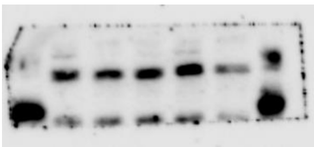	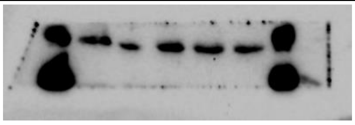
**Bax**	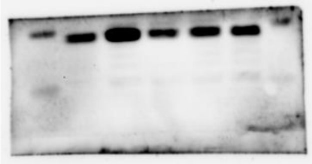	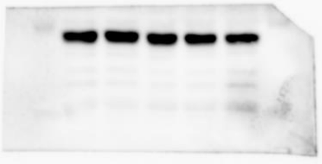	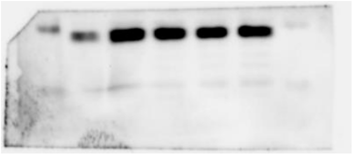

The sampling order of the blots is as follows: Control/Model/JWKXS-H/JWKXS-L/BYHWD. In addition to JWKXS, we actually provide BYHWD (Buyang Huanwu Decoction) to explore its mechanism. Due to some of its poor effects and unrelated to this article, it is not included. Original western blots were repeated three times.
