# Supplementary material for: Jia-Wei-Kai-Xin-San Treatment Alleviated Mild Cognitive Impairment through Anti-Inflammatory and Antiapoptotic Mechanisms in SAMP8 Mice
Source: Mediators Inflamm. 2023 Nov 2;2023:7807302. doi: 10.1155/2023/7807302 (PMC10635749; doi:10.1155/2023/7807302)
Supplement: Supplementary 2 — The sampling order of the blots is as follows: Control/Model/JWKXS-H/JWKXS-L/BYHWD. In addition to JWKXS, we actually provide BYHWD (Buyang Huanwu Decoction) to explore its mechanism. Due to some of its poor effects and unrelated to this article, it is not included. Original western blots were repeated three times. [file 7807302.f2.docx]

# Supplementary Figures and Tables

## Supplementary Fig.1


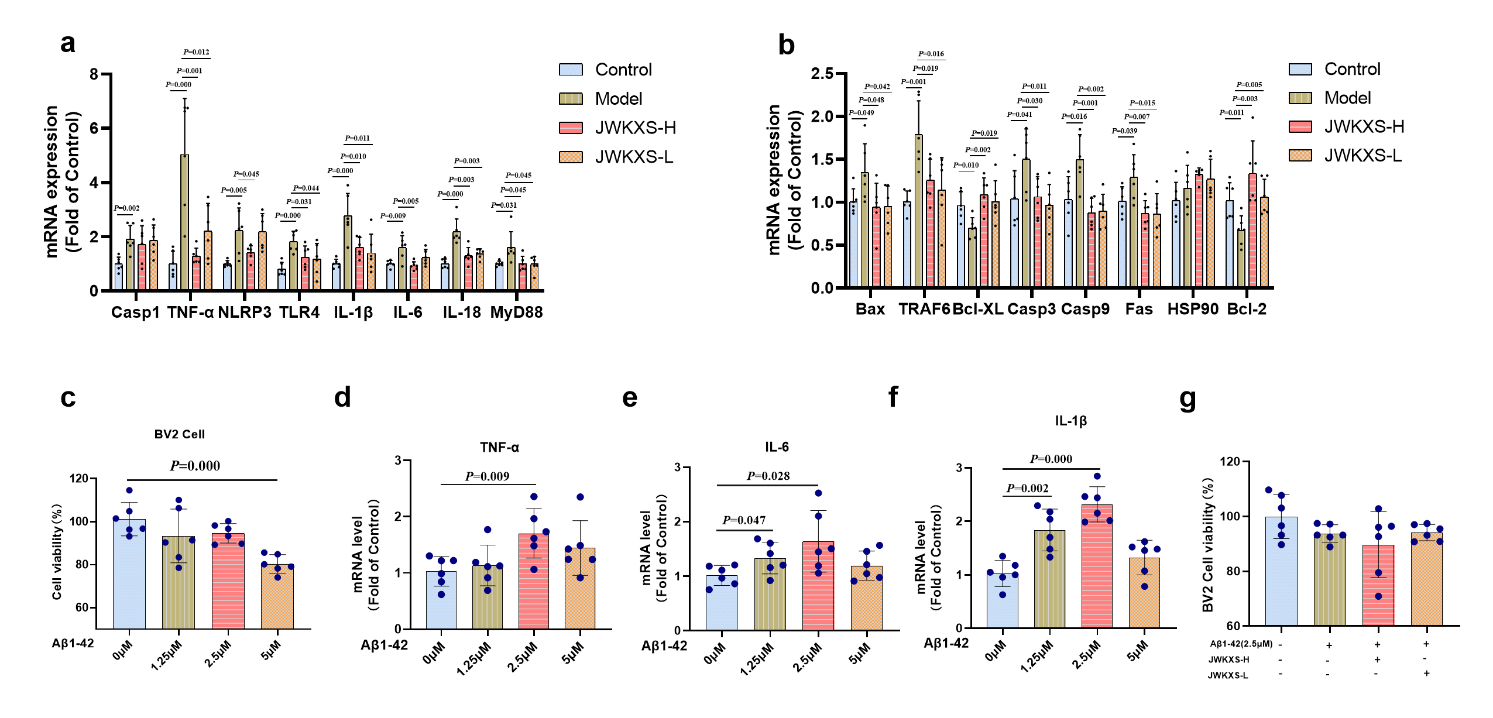


**Supplementary Fig. 1** (a-b) Transcription levels of genes involved in inflammation and apoptosis. (c, g) Cellular viability for BV2 microglial cells were treated or not treated with JWKXS. (d-f) mRNA expression of inflammatory cytokines TNF-α, IL-6, IL-1β in BV2 microglial cells intervened with different concentrations of Aβ1-42. Data were expressed as mean ± SD.

## Supplementary Tab.1

**Supplementary Tab. 1** Component analysis of JWKXS extract.

| **No.** | **Compound** | **Molecular formula** | **Error (PPM)** | **RT (min)** | **Measured Mass (m/z)** | **Mode** |
| --- | --- | --- | --- | --- | --- | --- |
| 1 | Campesteryl ferulate | C38H56O4 | -3.3 | 27.73 | 577.4257 | Pos |
| 2 | Nepetin | C16H12O7 | 1.3 | 15.69 | 317.0665 | Pos |
| 3 | MAV | C6H10O7 | 3.6 | 0.81 | 195.0505 | Pos |
| 4 | Ramalic acid | C18H18O7 | 7.8 | 5.57 | 347.1131 | Pos |
| 5 | Malic acid | C4H6O5 | 6.7 | 0.81 | 135.0293 | Pos |
| 6 | Tartaric acid | C4H6O6 | -3.3 | 7.42 | 151.0243 | Pos |
| 7 | Trifolin | C21H20O11 | -2 | 2.65 | 449.1084 | Pos |
| 8 | Xylose | C5H10O5 | -2.6 | 7.42 | 151.0606 | Pos |
| 9 | 25-Hydroxyprotopanaxatriol | C30H54O5 | 2.8 | 8.08 | 495.4049 | Pos |
| 10 | Oleuropein | C25H32O13 | -1.5 | 0.85 | 541.1921 | Pos |
| 11 | 12,13,15-Trihydroxy-9-octadecenoic acid | C18H34O5 | -3.9 | 26.12 | 331.2484 | Pos |
| 12 | Rhamnocitrin | C16H12O6 | 8.6 | 0.9 | 301.0712 | Pos |
| 13 | isorhamnetin | C16H12O7 | 1.3 | 15.69 | 317.0665 | Pos |
| 14 | (6aR,11aR)-9,10-dimethoxy-6a,11a-dihydro-6H-benzofurano[3,2-c]chromen-3-ol | C17H16O5 | -2.3 | 3.33 | 301.1069 | Pos |
| 15 | Arabinose,d | C5H10O5 | -8.6 | 9.05 | 151.0606 | Pos |
| 16 | homopolymer | C6H10O7 | 3.6 | 0.81 | 195.0505 | Pos |
| 17 | daidzein | 15H10O4 | -6.3 | 19.68 | 255.0641 | Pos |
| 18 | Mucronulatol | C17H18O5 | -5.9 | 2.21 | 303.1232 | Pos |
| 19 | Betulinic acid | C30H48O3 | -0.2 | 36.11 | 457.3682 | Pos |
| 20 | polygalaxanthone | C25H28O15 | -6 | 13.99 | 569.1506 | Pos |
| 21 | 2,5-Dihydroxy-6-methyl-7-methoxyflavanone | C17 H16 O5 | -4 | 3.33 | 301.1076 | Pos |
| 22 | 4-Methoxy-6-(11,12-methylenedioxy-14-methoxydihydrostyryl)-2-pyrone | C16 H16 O6 | 8.2 | 1.07 | 305.1025 | Pos |
| 23 | Onjixanthone II | C15 H12 O7 | -7.2 | 1.07 | 305.0661 | Pos |
| 24 | Crysophanol | C15H10O4 | -6.3 | 19.68 | 255.0657 | Pos |
| 25 | sucrose | C12H22O11 | -2.3 | 4.4 | 343.1232 | Pos |
| 26 | stearic acid | C18H36O2 | 5.3 | 27.4 | 285.2809 | Pos |
| 27 | (R)-p-Menth-1-en-4-ol | C10H18O | 0.6 | 25.85 | 155.1436 | Pos |
| 28 | Cistanoside H | C21H30O14 | 0 | 20.76 | 507.1714 | Pos |
| 29 | Salidroside | C14H20O7 | 9.6 | 0.8 | 301.1287 | Pos |
| 30 | Quercetin | C15H10O7 | -7.6 | 1.06 | 303.0505 | Pos |
| 31 | Heptane | C9H20 | -5.4 | 37.66 | 129.1643 | Pos |
| 32 | n-Butane | C4H10 | -8.5 | 17.06 | 59.0861 | Pos |
| 33 | 1-Triacontanol | C30H62O | 4.1 | 34.22 | 439.4879 | Pos |
| 34 | β-amyrin acetate | C32H52O2 | 9.2 | 28.42 | 469.4046 | Pos |
| 35 | L-Uridine | C_9_H_12_N_2_O_6_ | -4.9 | 0.82 | 245.0774 | Pos |
| 36 | Dihydroquercetin | C15H12O7 | -7.2 | 1.06 | 305.0661 | Pos |
| 37 | Tufulingoside | C15H16O9 | 5.3 | 8.44 | 341.0891 | Pos |
| 38 | 6α-Hydroxypolyporenic acid C | C31H46O5 | -7.2 | 27.14 | 499.3423 | Pos |
| 39 | Polyporenic acid C | C31H46O4 | 0 | 35.08 | 483.3474 | Pos |
| 40 | Pachymic acid | C33H52O5 | -0.4 | 26.69 | 539.3891 | Pos |
| 41 | Dehydrotrametenolic acid | C30H46O3 | 6.1 | 35.31 | 455.3525 | Pos |
| 42 | Dehydroeburicoic acid | C31H48O3 | 3 | 29.83 | 469.3628 | Pos |
| 43 | Eburicoic acid | C31H50O3 | -5.5 | 34.3 | 471.3838 | Pos |
| 44 | 1,6-Octadien-3-ol,3,7-dimethyl-, (3R)- | C10H18O | 0.6 | 25.85 | 155.1436 | Pos |
| 45 | Thymol | C10H14O | 6 | 22.08 | 151.1123 | Pos |
| 46 | Diethyl phthalate | C12H14O4 | -4 | 20.64 | 223.0961 | Pos |
| 47 | KAEMPFEROL-3-O-RUTINOSIDE | C27H30O15 | 5 | 9.13 | 595.1663 | Pos |
| 48 | Benzyl benzoate | C14H12O2 | 2.3 | 2.99 | 213.0916 | Pos |
| 49 | Bornyl acetate | C12H20O2 | 0 | 26.21 | 197.1542 | Pos |
| 50 | 2,5-Dimethoxybenzo-1,4-quinone | C8H8O4 | 5.3 | 10.06 | 169.0501 | Pos |
| 51 | aristol-9-en-8-one | C15H22O | 5 | 22.42 | 219.1749 | Pos |
| 52 | Benzyl chloride | C7H7Cl | -3.1 | 2.35 | 127.0315 | Pos |
| 53 | 2,4,5-Trimethoxybenzaldehyde | C10H12O4 | 7.1 | 16.96 | 197.0814 | Pos |
| 54 | Kaempferol | C15H10O6 | -6.3 | 1.06 | 287.0556 | Pos |
| 55 | 5-Hydroxymethylfurfural | C6H6O3 | 2.4 | 2.35 | 127.0395 | Pos |
| 56 | AZULENE | C10H8 | 8.5 | 5.92 | 129.0704 | Pos |
| 57 | Cinnamaldehyde | C9H8O | 9.8 | 0.94 | 133.0653 | Pos |
| 58 | Pentadecanoicacid | C18H32O2 | 0.4 | 31.71 | 281.2481 | Pos |
| 59 | Docosane | C22H46 | 8.7 | 26.42 | 311.3678 | Pos |
| 60 | Pentacosane | C25H52 | 6.5 | 35.79 | 353.4147 | Pos |
| 61 | ginsenoside Ro | C48H76O19 | 2.4 | 24.59 | 955.4903 | Neg |
| 62 | L-erythro-isocitric acid | C6H8O7 | -2.3 | 4.4 | 343.1232 | Neg |
| 63 | PANGAMIC ACID | C14H27NO8 | 7.4 | 0.77 | 336.1658 | Neg |
| 64 | Floralginsenoside Ta | C36H60O10 | -3.8 | 26.53 | 651.4108 | Neg |
| 65 | Dencichine | C5H8N2O5 | 0.6 | 5.82 | 175.0355 | Neg |
| 66 | Isomangiferin | C19H18O11 | -8.8 | 24.83 | 421.0771 | Neg |
| 67 | Phenylpropionic acid | C9H11NO2 | -0.6 | 3.07 | 164.0712 | Neg |
| 68 | Heriguard | C16H18O9 | -2.8 | 7.82 | 353.0863 | Neg |
| 69 | isoferulic acid | C10H10O4 | -3.6 | 12.69 | 193.0501 | Neg |
| 70 | formononetin | C16H12O4 | 3.4 | 24.66 | 267.0657 | Neg |
| 71 | GGB | C5H12N4O3 | 8 | 5.82 | 175.0831 | Neg |
| 72 | Crystal VI | C4H8N2O3 | 2.3 | 0.75 | 131.0457 | Neg |
| 73 | Cholest-5-en-3-ol | C30H52O | -0.5 | 20.99 | 427.394 | Neg |
| 74 | 3-Hydroxy-2-methylpyridine | C6H7NO | 4.6 | 19.18 | 108.0449 | Neg |
| 75 | irisxanthone | C20H20O11 | -3.9 | 13.82 | 435.0927 | Neg |
| 76 | 1-Hydroxy-3,6,7-trimethoxy xanthone | C16 H14 O6 | 9.3 | 0.9 | 301.0712 | Neg |
| 77 | Polygalasaponin XXVIII | C53H84O24 | 2.5 | 23.4 | 1103.5274 | Neg |
| 78 | Sitogluside | C35H60O6 | 7.6 | 27.61 | 575.4312 | Neg |
| 79 | tetradecane | C14H30 | -5.1 | 14.01 | 197.2269 | Neg |
| 80 | (+)-pinoresinol-O-β-D-glucopyranoside | C26H32O11 | -5 | 16.4 | 519.1866 | Neg |
| 81 | alexandrin | C35H60O6 | 7.6 | 27.61 | 575.4312 | Neg |
| 82 | Dehydrotumulosic acid | C31H48O4 | 0 | 35.08 | 483.3474 | Neg |
| 83 | Dehydropachymic acid | C33H50O5 | -6.3 | 33.83 | 525.358 | Neg |
| 84 | protocatechuic acid | C7H6O4 | -3.9 | 4.15 | 153.0188 | Neg |
| 85 | Majudin | C12H8O4 | -7.4 | 0.79 | 215.0344 | Neg |
| 86 | 2'-O-Methylisoliquiritigenin | C16H14O4 | 1.1 | 24.67 | 269.0814 | Neg |
| 87 | Emodin | C15H10O5 | 4.8 | 3.22 | 269.045 | Neg |
| 88 | 4-Hydroxycinnamic acid | C9H8O3 | 0.6 | 10.87 | 163.0396 | Neg |
| 89 | Linoleic acid | C15H30O2 | 2.1 | 28.44 | 241.2168 | Neg |
| 90 | Heneicosane | C21H44 | -6.4 | 9.71 | 295.3365 | Neg |
| 91 | Tetracosane | C24H50 | 3 | 19.51 | 337.3834 | Neg |

**2 Supplementary materials and methods**

**2.1** **Enzyme-linked immunosorbent assay (ELISA)**

The levels of NEFL, IL-6, TNF-α, IL-1β, and Aβ1-42 in the serum and hippocampus as well as the levels of IL-6, TNF-α, IL-1β in the cell supernatant were individually determined using ELISA kits, including a TNF-α, IL-1β, IL-6, NEFL and Aβ1-42 ELISA kit (jymbio, wuhan, China). All ELISA kits were used according to manufacturers' instructions, and hexafluoroethane was used to dissolve insoluble A1-42. The optical density values were measured 450 nm using a microplate reader.

**2.2 UPLC-Q-TOF/MS analysis**

The prepared JWKXS extracts (1.5 g/mL) were mixed with distilled water/acetonitrile (1: 1) to make a 20 mg/mL solution that was filtered through 0.22 μM filter membrane. Then, UPLC-Q/TOF/MS (Instrument: XEVO-G2QTOF#YCA345) was used to analyze 2 μL of the samples. The data were collected, and the chemical compositions of JWKXS were identified using UPLC-Q/TOF-MS technology and MSE mode. Chromatographic conditions: ACQUITY UPLC HSS T3 column (2.1×100 mm, 1.8 μm), the mobile phase contained distilled water (A)-acetonitrile (B), column temperature was set at 40℃. Gradient elution was used. Mass spectrometric conditions: the ESI-MS spectra were acquired in both positive ion voltage and negative ion voltage modes. The capillary voltage was set to 3.0 kV for the positive mode and to 2.5 kV for the negative mode, ESI ion source with temperature was set at 120℃, the desolvation temperature was 350℃, solvent-free gas flow rate was set at 300 L/h.

**2.3 Network pharmacology analysis**

TCMSP was used to identify all active compounds from the JWKXS decoction. The active compounds were screened according to oral bioavailability (OB) and druglikeness (DL). The PubChem and PharmMapper database were used to obtain the targets of active components in the JWKXS decoction. Similarly, MCI-related targets were obtained from OMIM and TTD databases. Then, the Venn diagram was used to cross the two targets of MCI and JWKXS to obtain the core JWKXS targets for treating MCI. The core targets were enriched with GO and KEGG pathways, and their effects of the core targets enriched on the key pathways were analyzed. When combined with the outcome effect analysis, the key regulatory targets and possible mechanisms of JWKXS for treating MCI were obtained.

**2.4** **RT‐qPCR**

Total RNA was extracted from the hippocampus or cell using Trizol and reverse transcribed into cDNA by Quantscript Reverse Transcriptase kit (Tiangen, China). The reverse transcribed products were amplified with Taq DNA polymerase (Tiangen, China). Primers were designed by Sangon Biotech (Shanghai, China) and cDNA was analyzed by qPCR on ABI 7500 system using UltraSYBR Mixture (CWBIO). Gene expression profile was analyzed by 2^−ΔΔCT^ method.

**2.5** **TUNEL staining**

Apoptotic cells were detected using TUNEL staining, performed with In Situ Cell Death Detection Kit (Roche, USA), as per the manufacturer’s instructions. The cells were analyzed by fluorescence microscope.

**2.6 Cell counting kit-8 (CCK-8) assay**

The BV2 microglial cells were seeded in 96-well plates and incubated in a complete medium. CCK-8 was added to a 96-well plate and incubated for 1 h. A wavelength of 450 nm was used to measure OD, and a curve was plotted based on the basis of OD values. The experiment was repeated three times in each group.

**2.7** **Assessment of Annexin V-FITC/PI Staining**

We evaluated apoptosis by flow cytometry using Annexin V-FITC/PI staining. After diving into differentiated PC12 cells were divided into different groups and incubating for 24 hours, the differentiated PC12 cells were harvested and stained with Annexin V-FITC and PI) for 5 min. Apoptosis was finally quantified using Flow cytometry.
